# LC-MS/MS Therapeutic Drug Monitoring of GS-441524 in Serum and Various Compounded Formulations to Improve the Treatment of Feline Infectious Peritonitis

**DOI:** 10.3390/ani16121851

**Published:** 2026-06-16

**Authors:** Riccardo Masti, Angela Marin, Luca Magna, Francesca Maria Bertolini, Tommaso Furlanello

**Affiliations:** San Marco Veterinary Clinic and Laboratory, Via dell’Industria 3, 35030 Veggiano, Italy; angela.marin@sanmarcovet.it (A.M.); luca.magna@sanmarcovet.it (L.M.); francesca.bertolini@sanmarcovet.it (F.M.B.)

**Keywords:** feline infectious peritonitis (FIP), GS-441524, ICH M10, serum amyloid A, surrogate matrix, 3Rs principles, therapeutic drug monitoring

## Abstract

Feline Infectious Peritonitis (FIP) has been transformed from a fatal disease to a treatable condition following the introduction of GS-441524, a nucleoside analogue targeting feline coronavirus replication. However, the widespread use of unregulated compounded formulations and the absence of validated analytical tools for therapeutic drug monitoring (TDM) represent critical gaps in clinical FIP management. This study describes the development and full ICH M10-compliant validation of a high-throughput LC-MS/MS method for the quantification of GS-441524 in feline serum, incorporating an automated protein precipitation protocol and a PBS-BSA surrogate matrix in accordance with 3Rs principles. The method met all acceptance criteria across validated parameters, including linearity (0.1–50 µg/mL), accuracy (bias within ±12.5%), precision (CV ≤ 10.9%), selectivity, extraction recovery (87.5–107.9%), and stability under clinically relevant storage conditions. Matrix equivalence between PBS-BSA and authentic feline serum was confirmed, enabling routine calibration without animal-derived materials. The validated method was applied to clinical TDM in cats undergoing GS-441524 treatment for FIP, providing preliminary evidence of inter-individual pharmacokinetic variability. The compounded formulations administered to the TDM cohort were independently verified by LC-MS/MS, confirming drug content within ±15% of labelled claims and excluding pharmaceutical quality as a confounding factor in the interpretation of serum drug concentrations.

## Simple Summary

Feline Infectious Peritonitis (FIP) is a viral disease of cats that, until recently, was invariably fatal. The introduction of GS-441524, an antiviral drug, has changed the prognosis, with remission rates exceeding 80%. However, most available preparations are compounded and unregulated, and limited LC-MS/MS validated methods exist for monitoring drug levels in treated cats. This study presents a fully validated method for measuring GS-441524 in feline serum, suitable for routine therapeutic drug monitoring. The method was applied to a cohort of cats undergoing FIP treatment, revealing marked differences in drug exposure between individuals. The compounded formulations used in the cohort were independently verified, confirming adequate drug content.

## 1. Introduction

Feline Infectious Peritonitis (FIP) is a systemic immunopathological disease caused by a virulent biotype of feline coronavirus (FCoV), designated as Feline Infectious Peritonitis Virus (FIPV), which selectively targets monocytes and macrophages [1,2]. For decades, FIP represented one of the most clinically challenging conditions in feline medicine, with a fatality rate approaching 100% [3]. The disease manifests across a spectrum of forms, ranging from the effusive type, characterised by vasculitis and exudative cavitary effusions, to non-effusive presentations involving granulomatous lesions, occasionally with neurological or ocular involvement with particularly guarded prognoses.

The therapy underwent a profound transformation with the characterisation of GS-441524 as a potent inhibitor of feline coronavirus (FCoV) replication, including the virulent FIPV biotype responsible for FIP. GS-441524 is a 1′-cyano-substituted adenine C-nucleoside ribose analogue and the active metabolite of remdesivir (GS-5734) [4,5,6]. Following intracellular phosphorylation, it is incorporated into nascent viral RNA chains as an alternative substrate for the viral RNA-dependent RNA polymerase, inducing delayed chain termination and halting viral replication [4,5]. Clinical studies and systematic reviews have reported overall treatment success rates of approximately 80–85%, with higher remission rates in effusive cases and more complex outcomes in neurological presentations [3,7]. The 2022 AAFP/Every Cat Feline Infectious Peritonitis Diagnosis Guidelines [8] and the European Advisory Board on Cat Diseases (ABCD) Guidelines [9] now recognise GS-441524 as a primary treatment option.

Despite its demonstrated efficacy, the regulatory availability of GS-441524 remains fragmented globally, and reliance on unlicensed compounded formulations raises significant concerns regarding product quality, concentration accuracy, and patient safety [10], underscoring the need for independent analytical verification tools. Fortunately, since October 2025, Italian veterinarians have had access to a legal source of compounded GS-441524, which is available for both oral and subcutaneous administration.

The clinical management of FIP is further complicated by the absence of robust pharmacokinetic (PK) and TDM data in cats. Existing quantification methods have primarily relied on HPLC-FLD, which offers limited sensitivity and selectivity in complex biological matrices and does not meet the requirements for rigorous clinical TDM [11]. A recent study provided valuable PK data following intravenous remdesivir administration and demonstrated interindividual variability in plasma trough concentrations across 22 clinical cases, explicitly highlighting the need for population pharmacokinetic modelling [12]. The recognition that individual cats may exhibit markedly different absorption profiles, particularly with oral formulations, further supports serum drug level monitoring as a tool for treatment personalisation and investigation of treatment failure [12,13].

In the absence of standardised pharmacodynamic endpoints, serum amyloid A (SAA) was integrated as a complementary marker of systemic inflammatory activity. SAA increases markedly during active FIP and decreases in response to effective antiviral treatment and has been proposed as a practical surrogate of treatment response [14]. Integrating SAA alongside drug quantification provides a complementary pharmacodynamic dimension to TDM data, potentially contextualising cases where serum GS-441524 concentrations fall below therapeutic targets.

Liquid chromatography–tandem mass spectrometry (LC-MS/MS) is an analytical technique in which analytes are first separated by their physicochemical properties on a chromatographic column and subsequently detected and quantified by a tandem mass spectrometer based on their mass-to-charge ratio. LC-MS/MS represents the gold standard for TDM in human and veterinary medicine [15,16,17,18], offering a combination of sensitivity, selectivity, and throughput suited to small-molecule antiviral quantification in complex matrices. The present study describes the development and full ICH M10 compliant validation [19] of an LC-MS/MS method for GS-441524 quantification in feline serum. In addition, the study investigates the equivalence between pooled feline serum and a surrogate matrix to minimise the use of biological material from healthy cats. It also evaluates the applicability of the method to TDM in cats undergoing FIP treatment, including assessment of the compounded formulations administered.

## 2. Materials and Methods

### 2.1. Serum Samples

Clinical Samples for Therapeutic Drug Monitoring

Serum samples were obtained from cats with confirmed feline infectious peritonitis (FIP) undergoing treatment with GS-441524. The diagnosis was based on clinical presentation, clinicopathological abnormalities (including hyperglobulinemia, hypoalbuminemia, and elevated acute-phase proteins) and confirmation of feline coronavirus infection by molecular methods (PCR) or immunocytochemistry. Samples were obtained 2–3 h post-dose (oral or injectable administration). Each cat contributed a single serum sample to the TDM cohort, and no longitudinal sampling was performed. Samples were collected opportunistically during routine clinical monitoring visits rather than at predefined study timepoints. Consequently, although all samples were obtained 2–3 h after drug administration, the duration of treatment at the time of sampling varied substantially among cats, ranging from 2 to 84 days. Clinical and pharmacological data were collected through a standardised informed consent form, recording signalment (age in months, body weight), FIP phenotype (effusive, non-effusive or with neurological involvement), formulation type (oral capsules, oral solution, or injectable), days on treatment at the time of sampling, daily dose, concomitant medications, and a subjective clinical efficacy assessment at the time of sampling (inadequate response, adequate response, or indeterminate). To assess the inflammatory status, serum amyloid A concentration with an immunoturbidimetric assay (LZ test SAA, Eiken Chemical Co., Tokyo, Japan) was measured.

Blood samples were collected from the jugular, cephalic, or saphenous veins using clot activator-free tubes (Becton-Dickinson, Franklin Lakes, NJ, USA). Samples were allowed to clot at room temperature for 30 min, then centrifuged at 3000× *g* for 10 min to separate serum, which was subsequently transferred to polypropylene microtubes and stored at −20 °C and analysed within 14 days after collection, consistent with the stability data established during method validation. For the matrix equivalence study, a pooled drug-free feline serum was prepared from residual samples obtained from six individual healthy adult cats presenting for routine health checks, with no clinical evidence of systemic disease and no known concurrent medications. Samples were confirmed to be GS-441524-free by analysis prior to pooling. For the selectivity assessment, the ten individual blank serum sources were also obtained from healthy cats. A single pool was used for matrix equivalence, consistent with standard practice under ICH M10.

All samples constituted residual biological material from routine diagnostic and monitoring procedures; no additional sampling was performed for research purposes. Written informed consent was obtained from all owners, including authorisation for the use of anonymized residual samples and clinical data for scientific publication. The study was conducted in compliance with Directive 2010/63/EU, Italian legislation (Decreto Legislativo 4 March 2014, n. 26), and institutional ethical guidelines.

### 2.2. Chemicals and Reagents

Reference Standards and Internal Standard

GS-441524 analytical standard (purity ≥ 98.0%) was purchased from AdipoGen Life Sciences (San Diego, CA, USA). 2-Chloroadenosine (purity ≥ 99.0%), used as an internal standard due to its similarity to GS-441524, was obtained from AdipoGen Life Sciences (San Diego, CA, USA). Both standards were stored as received at −20 °C under desiccated conditions and protected from light.

Solvents and Reagents

Methanol (LC-MS hypergrade, ≥99.9%) and acetonitrile (LC-MS grade) were purchased from LiChrosolv^®^ (Merck, Darmstadt, Germany). Dimethyl sulfoxide (DMSO, anhydrous, ≥99.9%) was obtained from Sigma-Aldrich^®^ (Merck, Darmstadt, Germany). Ammonium formate (≥99.0%, LC-MS grade) and formic acid (≥98%, LC-MS grade) were purchased from Sigma-Aldrich^®^ (Merck). Ultrapure water (18.2 MΩ·cm resistivity) was obtained from a Milli-Q^®^ water purification system (Merck Millipore, Burlington, MA, USA). PBS-BSA surrogate matrix was prepared by dissolving bovine serum albumin in phosphate-buffered saline (PBS, pH 7.4 sterile) (Sigma-Aldrich^®^) to a final concentration of 1% (*w*/*v*).

### 2.3. Instrumentation and Analytical Conditions

LC-MS/MS Instrumentation and Analytical Conditions

Chromatographic separation and mass spectrometric detection were performed using a Waters Acquity UPLC system coupled to a Waters TQ-S Micromass triple quadrupole mass spectrometer (Waters Corporation, Milford, MA, USA). Analyte separation was achieved on an Agilent Zorbax SB-C18 column (50 × 2.1 mm, 1.8 µm particle size; Agilent Technologies, Santa Clara, CA, USA) maintained at 45 °C. The mobile phase consisted of (A) ultrapure water containing 10 mM ammonium formate and 0.1% formic acid and (B) methanol, delivered at a flow rate of 0.3 mL/min. The gradient elution program was as follows: 0.0–0.5 min, 5% B (isocratic); 0.5–2.0 min, 5–50% B (linear gradient); 2.0–2.1 min, 50–100% B; 2.01–3.5 min, 100% B (column wash); 3.51 min, return to initial conditions (5% B) for re-equilibration. Total run time was 5.0 min per injection, with an injection volume of 5 µL.

The autosampler was maintained at 4 °C to preserve sample stability during batch analysis. The autosampler needle was rinsed with water/methanol (50:50, *v*/*v*) between injections to prevent carryover. The chromatographic conditions were optimized to achieve a retention time of approximately 1.8 min for GS-441524 and 2.1 min for the internal standard (2-chloroadenosine).

The mass spectrometer was operated in positive electrospray ionization (ESI+) mode with the following source parameters: capillary voltage, 1.0 kV; desolvation temperature, 650 °C; desolvation gas flow, 900 L/h; cone gas flow, 50 L/h. Multiple reaction monitoring (MRM) was employed for quantification and confirmation. For GS-441524, three MRM transitions were monitored: *m*/*z* 292.2 → 147.0 (quantifier; cone voltage 10 V, collision energy 30 eV), *m*/*z* 292.2 → 163.1 (qualifier 1; 10 V, 25 eV), and *m*/*z* 292.2 → 202.0 (qualifier 2; 10 V, 10 eV). The internal standard, 2-chloroadenosine, was monitored via two transitions: m/z 302.0 → 170.0 (quantifier; 10 V, 15 eV); *m*/*z* 302.0 → 134.0 (qualifier 1; 10 V, 40 eV). Dwell time for all transitions was 0.082 s to ensure adequate data point acquisition across the chromatographic peak (minimum 15 points per peak).

Ion ratio tolerances for qualifier transitions were established during validation and were required to fall within ±20% of the mean ratios calculated from calibration standards. Data acquisition and processing were performed using MassLynx software version 4.2 (Waters Corporation).

### 2.4. Preparation of Calibration Standards and Quality Control Samples

Calibration Curve and Solution Preparation

A primary stock solution of GS-441524 was prepared at a nominal concentration of 1.0 mg/mL using a mixture of acetonitrile and dimethyl sulfoxide (ACN/DMSO, 50:50, *v*/*v*). Similarly, an internal standard (IS) stock solution of 2-chloroadenosine was prepared at 1.0 mg/mL in ACN/DMSO (50:50, *v*/*v*) and subsequently diluted in pure methanol to a working concentration of 100 ng/mL, integrated directly into the protein precipitation reagent. The calibration curve, spanning a range from 0.1 to 50 µg/mL, was generated in the PBS-BSA surrogate matrix. To ensure high precision and minimize cumulative volumetric errors, all liquid handling and serial dilutions were automated using a Hamilton StarLET (Hamilton Company, Reno, NV, USA) liquid handling workstation. The automated dilution protocol utilized a systematic branching spanning a range from 0.1 to 50 µg/mL (Table S1).

### 2.5. Sample Preparation

Sample Extraction and Automated Processing

Sample extraction was performed using a one-step protein precipitation method (PPT) followed by dilution on the mentioned liquid handling workstation. The procedure was applied identically to calibration standards, quality control samples, and patient serum samples to ensure analytical consistency. Briefly, 50 µL of each sample were transferred into a 96-well collection plate (Phenomenex, Torrance, CA, USA). Protein precipitation was initiated by the addition of 450 µL of pure methanol containing the internal standard (2-chloroadenosine, 100 ng/mL). The plate was subjected to vigorous vortexing at 2500 rpm for 5 min to ensure complete protein denaturation, followed by centrifugation at 5000 rpm for 5 min at room temperature to achieve total sedimentation of the protein pellet. Post-centrifugation, the liquid handling workstation was utilized to carefully aspirate 50 µL of the clear supernatant, transferring it to a clean 96-well plate. This aliquot was subsequently diluted with 450 µL of a water/methanol mixture (50:50, *v*/*v*) containing 0.1% formic acid, resulting in an overall dilution factor of 1:100 from the original matrix.

### 2.6. Method Validation

The analytical method was validated according to the ICH M10 guideline on bioanalytical method validation and study sample analysis [19]. The validated quantification range spans 0.1–50 µg/mL.

#### 2.6.1. Calibration Curve Performance and Lower Limit of Quantification

Calibration curves were constructed by plotting the response of GS-441524 (y-axis) against the nominal concentration (x-axis) using weighted least-squares quadratic regression with a 1/y^2^ weighting factor. A quadratic model was selected over linear regression based on the observed non-linear detector response at higher concentrations, consistent with the known behaviour of triple quadrupole mass spectrometers operating across wide dynamic ranges. The 1/y^2^ weighting factor was applied to compensate for the increased variance at higher concentration levels and to ensure uniform accuracy across the full validated range [20]. The response function was assessed over three independent validation runs performed on separate days. Each calibration curve consisted of eight concentration levels analysed in triplicate. Acceptance criteria required a coefficient of determination (r^2^) ≥ 0.995 and back-calculated concentrations within ±15% of nominal values (±20% at LLOQ). The lower limit of quantification (LLOQ) was defined as the lowest concentration on the calibration curve (0.1 µg/mL) that could be quantified with acceptable accuracy (80–120%) and precision (CV ≤ 20%), with a signal-to-noise ratio ≥ 10:1.

#### 2.6.2. Accuracy and Precision

Intra-day accuracy and precision were evaluated by analysing five replicates of QC samples at four concentration levels (LLOQ-QC: 0.1 µg/mL, LQC: 0.3 µg/mL, MQC: 25 µg/mL, ULOQ-QC: 50 µg/mL) within a single analytical run. Inter-day accuracy and precision were assessed by analysing the same QC samples across three independent runs performed on separate days (total *n* = 15 per QC level). Accuracy was expressed as the percentage deviation of the mean measured concentration from the nominal concentration, calculated as: Accuracy (%) = (mean measured concentration/nominal concentration) × 100. Precision was expressed as the coefficient of variation (CV%), calculated as: CV (%) = (standard deviation/mean) × 100. Acceptance criteria were accuracy within 85–115% (80–120% at LLOQ) and precision CV ≤ 15% (≤20% at LLOQ).

#### 2.6.3. Matrix Effect and Extraction Recovery

Matrix effects were assessed using the post-extraction spike method in five different feline serum samples to evaluate the influence of endogenous matrix components on analyte ionization. Matrix effect was calculated as: Matrix Effect (%) = (Mean peak area of the analyte added post-precipitation/Mean peak area of the analyte in a neat analyte solution) × 100. A value of 100% indicates no matrix effect; values < 100% indicate ion suppression, and values > 100% indicate ion enhancement.

Extraction recovery was calculated as: Recovery (%) = (Mean peak area of extracted samples/Mean peak area of matrix-matched standards) × 100, representing the efficiency of the protein precipitation and dilution procedure.

#### 2.6.4. Carryover and Dilution Integrity

Carryover was assessed by injecting three blank samples immediately following the injection of samples at the upper limit of the calibration range (50 µg/mL). Carryover was considered acceptable if the peak area in the blank sample did not exceed 20% of the LLOQ response for the analyte and 5% for the internal standard.

Dilution integrity was assessed to demonstrate the accurate quantification of samples with concentrations exceeding the ULOQ. Five replicates of a quality control sample prepared at 200 µg/mL in PBS-BSA 1% were diluted 1:10 with blank PBS-BSA prior to extraction, yielding a theoretical concentration of 20 µg/mL in the matrix. Diluted samples were subsequently processed according to the standard sample preparation protocol described in Section 2.5. Back-calculated concentrations were obtained by applying a dilution factor of 10 to the measured values. Dilution integrity was considered acceptable when the mean back-calculated concentration remained within ±15% of the theoretical value of 200 µg/mL, with a precision (CV%) ≤ 15%.

#### 2.6.5. Selectivity

Selectivity was evaluated by analysing blank feline serum samples from ten individual cats to assess potential interference from endogenous components at the retention times and MRM transitions of GS-441524 and the internal standard. Samples were processed and analysed according to the sample preparation protocol described in Section 2.5. Additionally, these ten individual blank sources were spiked at the LLOQ level to evaluate the method’s performance in different matrices. The analytical response was considered acceptable if the accuracy was within ±20% of the nominal concentration.

#### 2.6.6. Stability Studies

The stability of GS-441524 in feline serum was evaluated using Low and Medium Quality Control samples (LQC: 0.3 µg/mL; MQC: 25 µg/mL) under four storage conditions: bench-top (20–25 °C), refrigerated (4 °C), frozen (−20 °C), and thermal stress (37 °C). Samples were analysed at days 1, 3, 7, and 15. Freeze–thaw stability was assessed by subjecting LQC and MQC samples to three consecutive freeze–thaw cycles, each consisting of storage at −20 °C for a minimum of 12 h followed by unassisted thawing at room temperature; a T0 reference sample was prepared in parallel and analysed prior to any freeze–thaw cycle. GS-441524 was considered stable when the mean back-calculated concentration remained within ±15% of the T0 reference value, with a precision (CV%) ≤ 15%.

Processed sample stability in the autosampler was assessed by comparing IS-normalized response ratios of extracted calibration standards maintained at 4 °C across three independent analytical runs performed on separate days. Stability was considered acceptable when the CV% of IS-normalized response ratios across runs did not exceed 15% (20% at LLOQ).

### 2.7. Pharmaceutical Quality Control Application

The GS-441524 content of compounded pharmaceutical formulations administered to cats included in the clinical TDM cohort was determined by LC-MS/MS, with the aim of excluding substandard drug quality as a potential confounding factor in the interpretation of serum drug concentrations. All formulations were sourced from compounding pharmacies authorised to operate in Italy. This analysis was performed independently of the bioanalytical method validated in feline serum, using a dedicated calibration curve prepared in pure solvent (water/methanol, 50:50, *v*/*v*, containing 0.1% formic acid) over a concentration range of 0.1–1 µg/mL. For each formulation, an accurately weighed amount of tablet powder or a precisely measured volume of oral solution was dissolved in ACN/DMSO (50:50, *v*/*v*). Serial dilutions were subsequently performed using the same solvent mixture to achieve concentrations within the calibrated range.

### 2.8. Statistical Analysis

Statistical analysis and graphical representation of data for the validation study were performed in Python 3.12 using NumPy 2.0 and SciPy 1.14 for numerical computations and Matplotlib 3.9 for data visualization [21,22,23]. Correlation between variables was assessed using Spearman’s rank correlation coefficient (ρ). Differences between two independent groups were evaluated using the Mann–Whitney U test, while comparisons among multiple groups were performed using the Kruskal–Wallis test. A two-tailed test was applied where relevant and *p*-values < 0.05 were considered statistically significant. The analysis of the TDM study were conducted using GraphPad Prism for MacOS version 9.5.1 (GraphPad Software, San Diego, CA, USA).

## 3. Results

### 3.1. Method Validation

**Figure 1:**
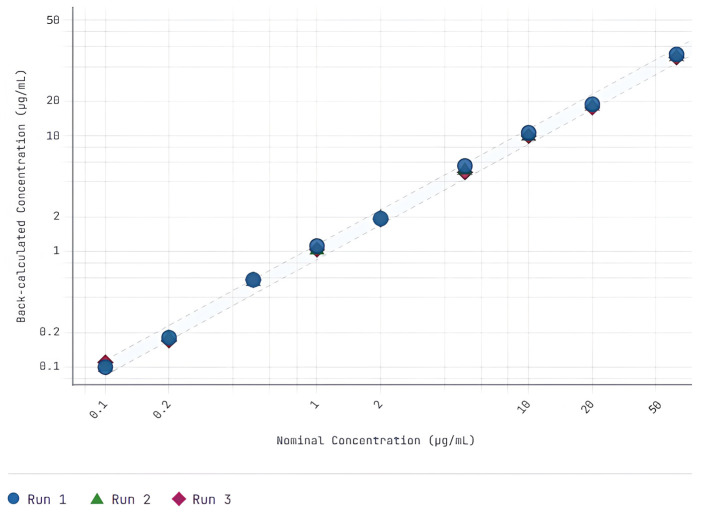
Back-calculated vs. nominal concentrations of GS-441524 across three independent analytical runs in PBS-BSA surrogate matrix (1% *w*/*v*). The dashed line represents the identity line (y = x). Data points represent individual calibration standards (0.1–50 µg/mL; *n* = 10 per run).

The analytical method was subjected to a comprehensive validation. The back-calculated accuracy for calibration standards ranged from 88.3% to 112.5%, meeting the regulatory requirement of a deviation within ±15% of the nominal value (±20% for the LLOQ). The demonstrated adequacy of the calibration model up to 50 μg/mL supports the reliable quantification of highly concentrated samples within the validated range (Figure 1).

Intra-day precision (CV%) ranged from 0.55% to 10.94%, with the highest variability observed at the LLOQ, as expected. Intra-day accuracy (Bias%) ranged from −12.5% to +7.9%. Inter-day precision, evaluated across three independent analytical sessions, ranged from 2.7% to 8.1%; inter-day accuracy ranged from −5.0% to +0.9%. All values fell within the pre-defined acceptance criteria of CV ≤ 15% and Bias within ±15% (≤20% and ±20% at the LLOQ, respectively).

Extraction recovery, assessed across all QC levels, ranged from 87.5% to 107.9%, confirming consistent and reproducible analyte isolation by the protein precipitation procedure across the validated concentration range (Table 1).

Selectivity was evaluated by analysing ten individual feline serum lots processed according to the standard sample preparation protocol. No interfering peaks were detected at the retention times of GS-441524 or the internal standard in any of the blank matrices, with endogenous signals below 20% of the LLOQ response for the analyte and below 5% for the internal standard.

### 3.2. Stability, Processed Sample Stability and Carryover

The study confirmed the stability of GS-441524 in feline serum across various storage conditions. Under frozen conditions, GS-441524 was stable at both levels throughout the entire 15-day period, with deviations ranging from −6.90% to +4.39% (Table 2). Under refrigerated conditions, stability was confirmed up to day 7 at both levels; at day 15, the LQC showed a deviation of −17.2%, marginally exceeding the acceptance threshold, while the MQC remained within criteria (+10.9%). At room temperature, stability was maintained up to day 7 at both levels, with failure observed at day 15 at both LQC (−24.1%) and MQC (−28.6%), indicating progressive analyte degradation upon prolonged storage at room temperature. Under thermal stress (37 °C), stability was maintained at days 1 and 3 but failed at day 7 for the LQC (−25.9%), while the MQC remained within criteria at day 7 (−14.7%); complete failure was observed at day 15 for both levels (LQC −46.6%, MQC −43.4%).

Freeze–thaw stability was confirmed at both concentration levels across all three cycles (Table 3). LQC samples showed deviations from T0 ranging from −6.9% (Thaw 2) to −3.4% (Thaw 1 and 3). MQC samples showed deviations ranging from −4.4% (Thaw 3) to +3.5% (Thaw 2). All values were well within the acceptance threshold of ±15%, confirming that GS-441524 in feline serum is stable through at least three freeze–thaw cycles under routine laboratory conditions.

Processed sample stability in the autosampler was assessed by comparing IS-normalized response ratios of extracted calibration standards maintained at 4 °C across three independent analytical runs performed on separate days (days 1, 4, and 7). CV% values ranged from 0.39% to 5.87% across all concentration levels, confirming adequate stability of processed samples under autosampler conditions for the duration of a typical analytical batch (Figure S1).

Carryover was evaluated by injecting blank samples immediately after the highest calibration standard. No significant signals were detected with areas consistently below 20% of the LLOQ, precluding risks of residual contamination between injections and confirming the solidity of the analytical system for high-throughput sequences. Dilution integrity was confirmed across five replicates at a nominal concentration of 200 µg/mL diluted 1:10. The mean back-calculated concentration was 215.2 µg/mL (accuracy: 107.6%, CV: 7.6%).

### 3.3. Matrix Equivalence and Surrogate Matrix Validation

**Figure 2:**
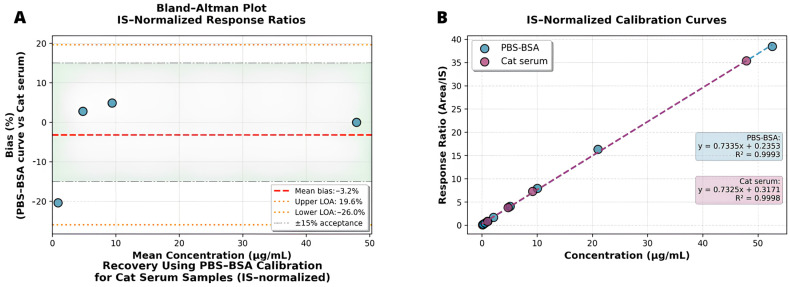
Matrix equivalence assessment of GS-441524 quantification using PBS-BSA as a surrogate for cat serum. All analyses were performed using IS-normalized response ratios (Area GS/Area IS). Bland–Altman plot showing agreement between PBS-BSA back-calculated and nominal cat serum concentrations (**A**). The green shaded area indicates the ±15% acceptance criteria. IS-normalized calibration curves for both matrices yielded virtually identical slopes (**B**).

Weighted quadratic regression (1/y^2^) of the IS-normalised response ratios yielded comparable curve parameters in both feline sera and surrogate matrices. The slope ratio between the two models was 0.93, indicating a differential matrix effect below 10% and therefore within acceptable limits. Matrix equivalence was further confirmed by back-calculating feline serum standards against the PBS-BSA calibration curve: all concentration levels demonstrated percentage deviations ranging from −4.0% to +3.2%, well within the regulated acceptance criteria of ±15% (±20% at the LLOQ). At the LLOQ specifically, the deviation was −1.3%, confirming adequate sensitivity and accuracy, even at the lower limit of the quantification range. IS-normalised matrix factors and extraction recovery were consistent across both matrices [24] (Figure 2).

### 3.4. Quality Control of Compounded GS-441524 Formulations from Commercial Sources

The GS-441524 content of five compounded pharmaceutical formulations administered to cats included in the clinical TDM cohort was determined by LC-MS/MS: three oral liquid preparations (nominal concentrations: 30 mg/mL and ~50 mg/mL) and two solid dosage forms (nominal content: 35 mg and 40 mg per unit). All five formulations demonstrated drug content within ±15% of the nominal concentration (Table S2).

### 3.5. Therapeutical Drug Monitoring

The validated LC-MS/MS method was applied to serum samples collected from seventeen cats undergoing GS-441524 treatment for confirmed FIP across multiple clinical presentations. The cohort included seventeen cats, with a median age of 11 months (range 6–108) and a median body weight of 3.0 kg (range 2.4–7.0) where available. The patients presented with wet (*n* = 7), dry (*n* = 5), and neurological/ocular (*n* = 5) forms of the disease, classified according to the predominant clinical presentation (Table S3). Formulations included injectable preparations, oral capsules and oral syrup. Days on treatment at the time of sampling ranged from 2 to 84 days, and daily doses ranged from 6 to 50 mg/kg, in accordance with the 2022 AAFP/EveryCat Feline Infectious Peritonitis Diagnosis Guidelines and the European Advisory Board on Cat Diseases (ABCD) recommendations [8,9].

**Figure 3:**
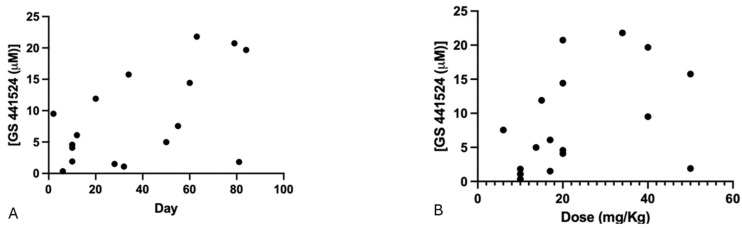
Positive correlation between serum GS-441524 and day of treatment (**A**), and serum GS-441524 and administered dose (**B**).

**Figure 4:**
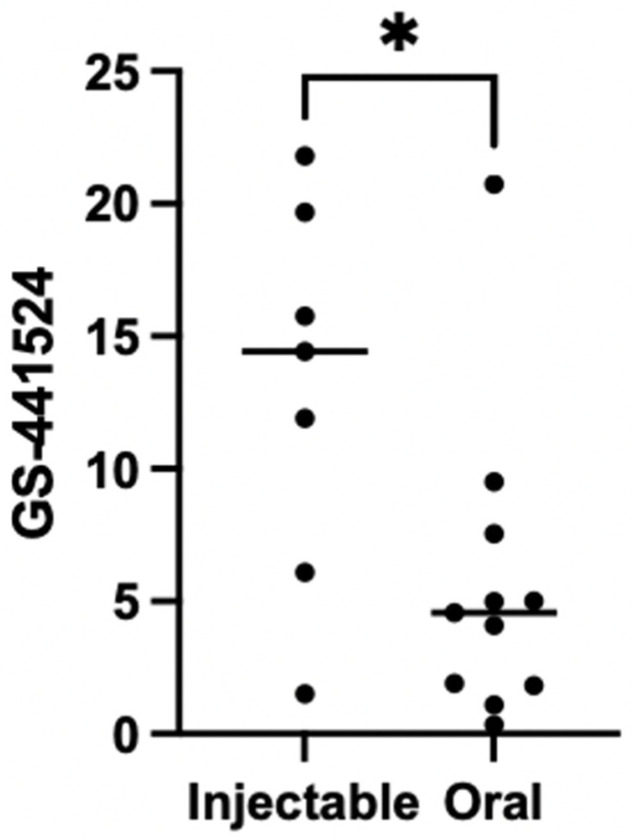
Comparison of serum GS-441524 concentrations in cats undergoing oral vs. injectable administration. * Statistically significant difference between groups (*p* < 0.05).

**Figure 5:**
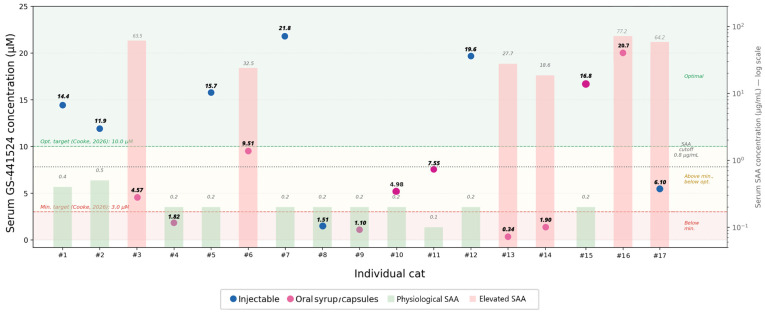
Serum GS-441524 concentrations in seventeen FIP-treated cats, classified by formulation type. Dashed lines: minimum (3.0 µM) and optimal (10.0 µM) therapeutic targets as proposed by Cooke et al. [13]. Coloured zones: below minimum (red), above minimum but below optimal (yellow), optimal range (green). Samples collected 2–3 h post-dose. Bars: concurrent SAA concentrations (right y-axis, log scale, µg/mL); green = within physiological range (≤0.8 µg/mL), red = above upper reference limit.

**Figure 6:**
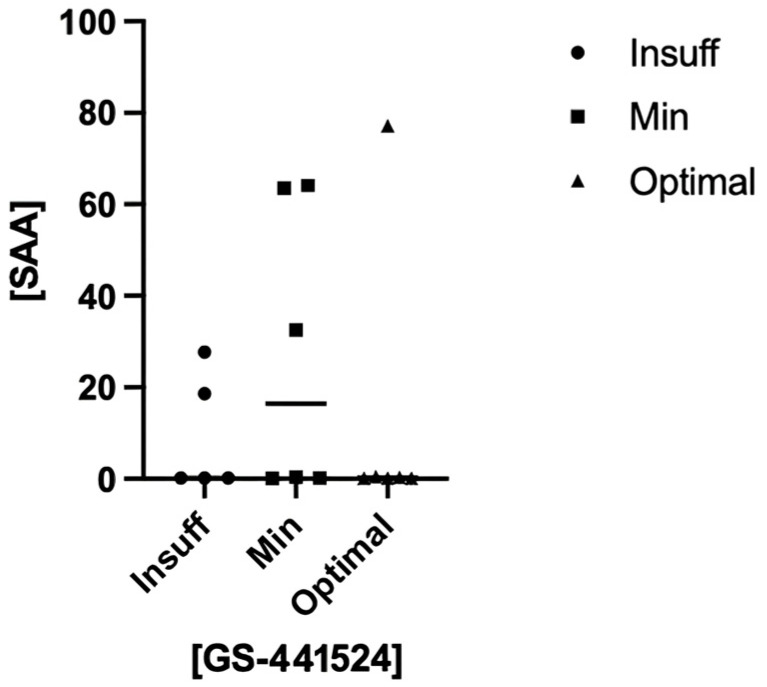
SAA concentrations in cats grouped according to therapeutic target ranges.

Serum GS-441524 concentrations ranged from 0.34 to 21.80 µM across the cohort, with a median of 5.0 µM, reflecting marked inter-individual variability. When classified according to the therapeutic targets proposed by Cooke et al. [13], a minimum target of >3.0 µM and an optimal target of >10.0 µM, six cats (35.3%) achieved optimal concentrations (>10.0 µM), five cats (29.4%) showed concentrations above the minimum but below the optimal threshold (3.0–10.0 µM), and six cats (35.3%) fell below the minimum therapeutic target (<3.0 µM). Serum GS-441524 concentrations positively correlated with duration of treatment (r = 0,49; *p* = 0.04; CI = 0.01–0.79) and with the administered dose (r = 0.52; *p* = 0.03; CI = 0.03–0.8), (Figure 3). When comparing the route of administration, serum GS-441524 was significantly higher in cats treated with injectable formulations compared to oral formulations (*p* = 0.04) (Figure 4). Concurrent SAA concentrations were available for all cats in the cohort and ranged from 0.1 to 77.2 µg/mL (reference interval: 0.1–0.8 µg/mL). SAA concentrations within the reference limits during treatment were observed in twelve cats (70.6%), including three cats whose serum GS-441524 concentrations fell below the minimum therapeutic target of 3.0 µM. (Figure 5). Additionally, SAA levels did not correlate with serum GS -441524 concentrations and were not significantly different among cats stratified by GS-441524 therapeutic target ranges (Figure 6). Finally, serum GS-441524 concentrations were not statistically different in cats grouped according to clinical presentation of FIP (wet and dry).

## 4. Discussion

The present study pursued two primary objectives: the development and analytical validation of a high-throughput LC–MS/MS method for the quantification of GS-441524 in feline serum, and the preliminary application of the validated method to clinical TDM in cats with FIP. In parallel, the LC-MS/MS platform was employed for the independent analytical verification of compounded GS-441524 formulations from multiple legal sources. These objectives were integrated to provide a robust analytical and clinical framework, with the prospect of moving beyond empirical dosing toward a patient-specific approach in feline antiviral therapy.

A significant analytical contribution of this study concerns the validation of PBS-BSA (1%) as a surrogate matrix for calibration. Previous methods have relied on authentic blank feline serum for matrix matching [11], inherently limited by biological variability between donor lots and the ethical constraints associated with sourcing large volumes of feline blood. The use of a protein-matched surrogate matrix, coupled with a protein precipitation sample preparation, effectively normalizes the chemical environment of the feline sample, mirroring human bioanalytical protocols where authentic matrix standardization is critical [25]. Cross-validation between the surrogate and authentic feline matrices confirmed overlapping response functions, eliminating the need for animal-derived materials in accordance with the 3Rs principles (replacement, reduction, refinement of animal use in research) while enhancing inter-laboratory reproducibility [26].

Compared to traditional chromatographic methods (HPLC-UV/FLD) [11], which may be susceptible to endogenous interferences in metabolically deranged patients, this LC–MS/MS protocol provides superior molecular selectivity through the monitoring of specific mass-to-charge transitions [17]. Accuracy values reflect overall method performance including extraction recovery and matrix effects, indirectly supporting the effectiveness of the protein precipitation procedure. Selectivity was assessed across 10 individual feline sera, ensuring that biological variability does not compromise diagnostic accuracy. 2-Chloroadenosine was selected as internal standard based on its structural similarity to GS-441524 and chromatographic compatibility [11,27]. The method was validated over a wide calibration range (0.1–50 µg/mL), encompassing concentrations well above those typically reported in feline literature (~8.7 µg/mL) [12], with dedicated carryover evaluation, thereby eliminating the need for sample dilutions and reducing pre-analytical error. A weighted quadratic regression model (1/y^2^) was applied to compensate for the non-linear detector response at higher concentrations characteristic of triple quadrupole systems, ensuring optimal accuracy throughout the analytical range in accordance with established bioanalytical best practices [20].

GS-441524 demonstrated acceptable stability for up to 15 days at −20 °C, 7 days at 4 °C and 20–25 °C, and 3 days at 37 °C, supporting flexible sample handling and inter-laboratory transport. Frozen storage at −20 °C represents the preferred condition for long-term preservation, while refrigerated and room-temperature storage should be limited to one week.

A fundamental prerequisite for the valid pharmacokinetic interpretation of TDM data is the exclusion of pharmaceutical quality as a confounding variable, as serum concentration deviations may reflect patient pharmacokinetics or inconsistent drug content. In the context of compounded veterinary preparations, where manufacturing lacks the regulatory oversight applied to licensed products, this distinction must be empirically verified [10]. Independent LC-MS/MS quantification of the five formulations used in the TDM cohort confirmed drug content within ±15% of labelled claims for all preparations. Critically, the cat exhibiting the lowest serum GS-441524 concentration received a formulation whose drug content was independently verified as adequate, allowing the observed exposure deficit to be attributed to patient-level pharmacokinetic factors rather than to a product quality failure. The lower recovery observed for the 40 mg tablet likely reflects matrix-dependent extraction efficiency, where excipient interactions may modulate analyte release during solvent dissolution; future studies employing sonication-assisted or sequential solvent extraction are warranted for this dosage form. The analysis presented here should be regarded as an exploratory assessment of content adequacy, rather than a validated protocol for pharmaceutical batch release [10,13].

The preliminary clinical application of the validated method revealed marked inter-individual variability in serum GS-441524 concentrations, ranging from 0.34 to 21.80 µM across the cohort. Cats receiving injectable formulations consistently achieved concentrations within the optimal therapeutic range proposed by Cooke et al. [13], whereas cats treated with oral formulations exclusively showed concentrations predominantly below or at the lower boundary of the therapeutic target. This observation is consistent with the known pharmacokinetic limitations of oral nucleoside analogue delivery and may reflect several concurring mechanisms.

Although GS-441524 is structurally resistant to acid hydrolysis, rendering gastric degradation an unlikely primary cause of reduced oral bioavailability, intestinal absorption represents a critical bottleneck. As a polar molecule with low lipophilicity, GS-441524 relies on carrier-mediated transport across the intestinal epithelium. Inter-individual variability in the expression and activity of these transporters may substantially influence the fraction of drug absorbed, contributing to the wide concentration range observed in orally treated cats [13]. Following intestinal absorption, hepatic first-pass metabolism represents an additional source of variability, particularly in cats with FIP-associated hepatic involvement, where compromised liver function may alter metabolic clearance unpredictably. Beyond intrinsic pharmacokinetic factors, several extrinsic variables may have contributed to the observed variability. Although in all cases the compounded drug was administered under veterinary prescription, indicating fasting at the time of administration, food intake is known to modulate nucleoside transporter competition and gastric pH, both of which influence oral drug absorption. Owner-administered oral formulations introduce additional uncertainty regarding compliance and completeness of dosing, as partial loss during administration, excessive salivation, or prompt regurgitation cannot be excluded. Concomitant medications may further modulate gastrointestinal physiology, corticosteroids can alter nucleoside transporter expression, while prokinetic or antiemetic agents may modify intestinal transit time, narrowing the absorption window [13]. Finally, the volume of distribution of GS-441524 may be substantially increased in cats with active FIP and significant body cavity effusions, as drug redistribution into effusion fluid may dilute systemic concentrations independently of the absorption route. However, in our study, although serum GS-441524 was lower in cats with wet FIP compared to dry FIP, the difference was not significant.

Although a positive correlation between serum GS-441524 and dose was observed, collectively, these findings underscore the multifactorial nature of pharmacokinetic variability in GS-441524 therapy and highlight the inadequacy of dose alone as a predictor of systemic drug exposure. Interpretation of the relationship between dose and systemic exposure should take into account that dosing recommendations are not uniform across formulations and routes of administration. While higher dosages are generally recommended for oral GS-441524 because of its lower and more variable bioavailability, parenteral administration has been described in previous studies and clinical practice using lower dosing regimens, which may still achieve adequate systemic exposure due to improved bioavailability [7]. Concurrent SAA measurements provided a complementary pharmacodynamic dimension to TDM data: SAA values within the reference interval were observed in twelve cats (70.6%), including three cats whose serum GS-441524 concentrations fell below the minimum therapeutic target of 3.0 µM. This apparent dissociation between subtherapeutic drug exposure and suppressed inflammatory activity suggests that the relationship between serum GS-441524 concentration and pharmacodynamic response may not be strictly linear, and that some individuals may achieve adequate viral suppression at concentrations below the proposed thresholds. Conversely, six cats with pathologically elevated SAA concentrations received formulations whose drug content was independently verified as adequate, indicating that inadequate treatment response in these cases was attributable to pharmacokinetic rather than pharmaceutical factors. In addition, concurrent inflammatory processes not strictly correlated with FIP virus infection may confound the interpretation of serum SAA concentrations. For example, patient 17 developed painful subcutaneous lesions at the injection sites that subsequently ulcerated (Figure S2), plausibly eliciting a secondary inflammatory process that may have influenced serum SAA concentrations independently of FIP-related inflammation.

### Limitations

Several limitations of the present study warrant consideration. The relatively small sample size precludes the establishment of definitive therapeutic reference ranges, and TDM interpretation remains inherently complex due to variability in sampling timing, individual pharmacokinetics, and patient clinical status. Serum concentrations were evaluated exclusively at the expected peak, yet the precise timing of sample collection relative to drug administration was not systematically recorded, introducing uncertainty in the pharmacokinetic interpretation. An additional limitation is the variability in treatment duration at the time of sampling. Because cats were sampled at different stages of therapy, measured serum concentrations may reflect different pharmacokinetic conditions. The moderate positive correlation observed between serum GS-441524 concentration and treatment day suggests that treatment duration may partially contribute to the observed inter-individual variability in systemic exposure. Standardisation of sampling timepoints relative to treatment initiation and assessment under steady-state conditions should therefore be considered in future pharmacokinetic–pharmacodynamic investigations.

Despite these limitations, to our knowledge, this is the first study to investigate GS-441524 TDM encompassing legally available oral formulations in naturally infected cats, which represents a meaningful contribution to the field. Nevertheless, larger prospective pharmacokinetic–pharmacodynamic studies correlating systemic drug exposure with clinically meaningful outcomes will be essential to advance toward evidence-based dosing strategies [12,13].

Regarding analytical performance, long-term specimen stability beyond twelve months at −20 °C was not assessed in the present work; future investigations should address this to support systematic biobanking initiatives. That said, the stability demonstrated under standard freezer conditions (−20 °C) ensures broad applicability in routine veterinary diagnostic settings where ultra-low temperature storage may not be available.

With respect to disease monitoring, α-1-acid glycoprotein (AGP) is currently considered the most informative acute-phase protein for FIP follow-up [9]; however, SAA has also been used [14] and it was selected in the present study on account of its lower cost and wider accessibility in clinical practice. The relationship between AGP dynamics and GS-441524 systemic exposure merits dedicated investigation in future studies.

Finally, adequate central nervous system penetration is a critical determinant of therapeutic success in neurological FIP. Future studies should evaluate GS-441524 concentrations not only in cerebrospinal fluid in cats with neurological FIP, but also in aqueous humour in cases with ocular involvement, using the validated method described herein. Characterisation of drug distribution within these compartments, together with compartmental pharmacokinetic modelling, would contribute to refining dosing regimens for these challenging clinical presentations. [28].

## 5. Conclusions

The present study establishes a robust, high-throughput LC-MS/MS method for the quantification of GS-441524, meeting the dual requirements of pharmaceutical quality control and clinical TDM. The validation of a surrogate matrix approach ensured high inter-laboratory reproducibility by reducing the biological variability inherent in authentic serum, while aligning with 3Rs principles.

Validated according to ICH M10 guidelines, this LC-MS/MS method enables precise GS-441524 quantification to guide evidence-based dosing strategies for FIP management. The clinical application of the method confirmed the acceptable quality of the tested compounded formulations while simultaneously highlighting the critical role of individual pharmacokinetic variability.

As GS-441524 continues to transform the prognosis of FIP from a uniformly fatal disease to a manageable and treatable condition, the systematic application of validated TDM emerges as a valuable tool for optimizing individual patient care and reduce the number of non-responding cats, even in the minority of cases that do not respond to standard dosing [9]. By enabling personalized drug monitoring, this method supports standardized dosing protocols and the development of evidence-based veterinary therapeutics.

## Figures and Tables

**Table 1:** Intra- and inter-assay precision and accuracy of GS-441524 in feline serum at four quality control levels. Intra-assay parameters were evaluated across three independent analytical runs. Inter-assay parameters were calculated across all three runs. Acceptance criteria: CV ≤ 15% (≤20% at LLOQ); accuracy within ±15% (±20% at LLOQ); bias within 85–115% (80–120% at LLOQ).

Spike Level	Parameter	Intra-Assay			Inter-Assay
		Day 1	Day 2	Day 3	
LLOQ-QC (0.1 µg/mL)	Mean (µg/mL)	0.09	0.10	0.10	0.10
	CV (%)	10.9	9.82	0.00	7.00
	Bias (%)	−12.5	−2.50	0.01	−5.00
	Accuracy (%)	87.5	97.5	100.0	95.0
LQC (0.3 µg/mL)	Mean (µg/mL)	0.29	0.31	0.30	0.30
	CV (%)	3.04	3.23	2.94	2.70
	Bias (%)	−2.00	3.30	1.30	0.90
	Accuracy (%)	98.0	103	101	101
MQC (25.0 µg/mL)	Mean (µg/mL)	22.9	25.1	25.1	24.4
	CV (%)	1.82	1.49	0.55	5.30
	Bias (%)	−8.50	0.40	0.40	−2.60
	Accuracy (%)	91.5	100	100	97.4
HQC (50.0 µg/mL)	Mean (µg/mL)	47.1	53.9	47.1	49.4
	CV (%)	3.55	2.05	3.55	8.10
	Bias (%)	−5.90	7.90	−5.90	−1.30
	Accuracy (%)	94.1	107.9	94.1	98.7

LLOQ: lower limit of quantification; LQC: low quality control; MQC: medium quality control; HQC: high quality control.

**Table 2:** Stability of GS-441524 in feline serum under four storage conditions assessed at days 1, 3, 7, and 15. Results are expressed as mean measured concentration and percentage deviation relative to T0 for LQC (0.3 µg/mL) and MQC (25 µg/mL) samples. ✓: within ±15% of T0 (acceptance criterion); ✗: exceeds acceptance criterion.

Timepoint/ Condition	LQC				MQC			
	Mean	SD	% Resp. Relative to T0	Acceptability	Mean	SD	% Resp. Relative to T0	Acceptability
T0	0.29	0.01	—	—	26.8	0.11	—	—
Benchtop day 1	0.28	0.01	−5.17%	✓	27.1	0.04	0.95%	✓
Benchtop day 3	0.30	0.01	1.72%	✓	28.1	1.44	4.72%	✓
Benchtop day 7	0.27	0.01	−8.62%	✓	25.8	0.42	−3.88%	✓
Benchtop day 15	0.22	0.03	−24.1%	✗	19.1	0.63	−28.6%	✗
Refrigerated day 1	0.29	0.01	0.00%	✓	26.8	0.11	0.11%	✓
Refrigerated day 3	0.31	0.01	6.90%	✓	29.0	1.03	8.29%	✓
Refrigerated day 7	0.26	0.01	−10.3%	✓	27.2	0.62	1.44%	✓
Refrigerated day 15	0.24	0.01	−17.2%	✗	24.4	0.24	10.9%	✓
Frozen day 1	0.28	0.01	−3.45%	✓	27.4	0.05	2.35%	✓
Frozen day 3	0.30	0.01	1.72%	✓	28.0	0.25	4.39%	✓
Frozen day 7	0.29	0.01	−1.72%	✓	27.4	0.47	2.20%	✓
Frozen day 15	0.27	0.01	−6.90%	✓	24.5	0.18	−8.45%	✓
Thermal stress day 1	0.29	0.01	−1.72%	✓	25.5	0.40	−4.93%	✓
Thermal stress day 3	0.28	0.01	−3.45%	✓	27.1	0.33	1.19%	✓
Thermal stress day 7	0.22	0.02	−25.9%	✗	22.9	0.53	−14.7%	✓
Thermal stress day 15	0.16	0.01	−46.6%	✗	15.2	0.03	−43.4%	✗

SD: standard deviation. LQC nominal: 0.3 µg/mL; MQC nominal: ~27 µg/mL.

**Table 3:** Freeze–thaw stability of GS-441524 in feline serum across three consecutive freeze–thaw cycles. Results are expressed as mean measured concentration and percentage deviation relative to T0 for LQC (0.3 µg/mL) and MQC (~27 µg/mL) samples. ✓: within ±15% of T0 (acceptance criterion).

Timepoint/ Condition	LQC				MQC			
	Mean	SD	% Resp. Relative to T0	Acceptability	Mean	SD	% Resp. Relative to T0	Acceptability
T0	0.29	0.00	—	—	27.4	0.34	—	—
Thaw 1	0.28	0.00	−3.40	✓	28.2	0.53	3.00	✓
Thaw 2	0.27	0.00	−6.90	✓	28.4	0.31	3.50	✓
Thaw 3	0.28	0.00	−3.40	✓	26.2	0.14	−4.40	✓

SD: standard deviation. LQC nominal: 0.3 µg/mL; MQC nominal: ~27 µg/mL.

## Data Availability

The dataset is available on request from the authors due to patient and owner privacy restrictions.

## Supplementary Materials

The following supporting information can be downloaded at: https://www.mdpi.com/article/10.3390/ani16121851/s1, Table S1. Detailed serial dilution protocol for GS-441524 calibration standards. Table S2. LC-MS/MS quantification of GS-441524 content in compounded formulations administered to the therapeutic drug monitoring cohort. Table S3. Clinical and pharmacological characteristics of cats undergoing GS-441524 TDM. Figure S1. Processed sample stability assessed by CV% of IS-normalized response ratios across three independent analytical runs (days 1, 4, and 7) for extracted calibration standards maintained at 4°C in the autosampler. Figure S2. Ulcerated subcutaneous lesions at the injection site in cat 17.
