# Supplementary material for: LC-MS/MS Therapeutic Drug Monitoring of GS-441524 in Serum and Various Compounded Formulations to Improve the Treatment of Feline Infectious Peritonitis
Source: Animals (Basel). 2026 Jun 16;16(12):1851. doi: 10.3390/ani16121851 (PMC13296256; doi:10.3390/ani16121851)
Supplement: Supplementary file 1 [file animals-16-01851-s001.zip › animals-4325631-supplementary.pdf]

animals

MDPI

Article

LC-MS/MS Therapeutic Drug Monitoring of GS-441524 in Serum and Various Compounded Formulations to Improve the Treatment of Feline Infectious Peritonitis

Riccardo Masti *, Angela Marin, Luca Magna, Francesca Maria Bertolini and Tommaso Furlanello *

San Marco Veterinary Clinic and Laboratory, Via dell'Industria 3, 35030 Veggiano, Italy; angela.marin@sanmarcovet.it (A.M.); luca.magna@sanmarcovet.it (L.M.); francesca.bertolini@sanmarcovet.it (F.M.B.)
* Correspondence: riccardo.masti@sanmarcovet.it (R.M.); tf@sanmarcovet.it (T.F.)

Supplementary

Table S1. Detailed serial dilution protocol for GS-441524 calibration standards. All dilutions were performed using an automated liquid handling workstation (Hamilton StarLET) to ensure precise volumetric dispensing and to minimize cumulative dilution errors. Concentrations represent the final values in the surrogate matrix (PBS-BSA 1%).

Level	Final Conc. (μg/mL)	Source Solution	Source Vol. (μL)	Diluent Vol. (μL)	Total Vol. (μL)	Ratio
WS1	100	Primary Stock	100	900	1000	01:10
WS2	50	Primary Stock	50	950	1000	01:20
WS3	20	WS1	200	800	1000	01:05
WS4	10	WS1	100	900	1000	01:10
WS5	5	WS2	100	900	1000	01:10
WS6	2	WS3	100	900	1000	01:10
WS7	1	WS4	100	900	1000	01:10
WS8	0.5	WS5	100	900	1000	01:10
WS9	0.2	WS6	100	900	1000	01:10
WS10	0.1	WS7	100	900	1000	01:10
WS11	0.1	WS8	100	900	1000	01:10

WS, Working Standard; Conc., concentration; Vol., volume.

Table S2. LC-MS/MS quantification of GS-441524 content in compounded formulations administered to the therapeutic drug monitoring cohort.

Formulation	Nominal content	Expected conc. (ng/mL)	Found conc. (ng/mL)	Individual recovery (%)	Found content	Mean recovery (%)	Result
Oral syrup 30 mg/mL	30 mg/mL	300 / 30 / 3	314.5 / 34.1 / 3.4	104.8%	33.2 mg/mL	110.7%	✓
Tablet 40 mg	40 mg/unit	200 / 20 / 2	193.5 / 18.2 / 1.7	90.9%	36.4 mg/unit	90.90%	✓
Tablet 35 mg	35 mg/unit	350 / 35 / 3.5	356.1 / 36.6 / 3.8	101.7%	36.7 mg/unit	105.0%	✓
Oral syrup 50 mg/mL	50 mg/mL	500 / 50 / 5	423.3 / 45.9 / 4.9	84.7%	46.1 mg/mL	91.5%	✓
Oral syrup 30 mg/mL (2nd source)	30 mg/mL	300 / 30 / 3	294.0 / 32.6 / 3.6	98.0%	32.8 mg/mL	108.90%	✓

Recovery (%): found/expected × 100. Acceptance criterion: 85–115% of nominal (±15%). Mean recovery calculated across all measured dilutions per formulation. ✓: all dilutions within acceptance criteria.

Table S3. Clinical and pharmacological characteristics of cats undergoing GS-441524 TDM.

Case	Sex	BW (kg)	Age (months)	FIP type	Formulation	Days on treatment	Dose (mg/kg)	GS-441524 (µM)	SAA (µg/ml)	Clinical status	Comments
1	MC	2.6	6	Neurological + Ocular	Injectable	60	10	14.4	0.4	Adequate	Resolution of neurological but not ocular form
2	MC	4.4	94	Dry	Injectable	20	6	11.9	0.5	Adequate	
3	FOHE	ND	60	Dry + Neurological	Capsules	10	20	4.57	63.5	Unsure	
4	FI	ND	8	Wet	Capsules	81	50	1.82	0.2	Adequate	
5	FI	2.7	8	Dry + Neurological	Injectable	34	10	15.8	0.2	Adequate	
6	MC	4.8	11	Wet	Oral syrup	2	50	9.51	32.5	Unsure	
7	MC	5	67	Wet	Injectable	63	40	21.8	0.2	Adequate	BW increase 3.4→5.0 kg; resumed eating
8	FOHE	5	36	Wet	Injectable	28	34	1.51	0.2	Adequate	Injectable route; mild neutropenia
9	MC	ND	9	Dry	Oral syrup	32	20	1.1	0.2	Adequate	
10	MI	7	20	Wet	Capsules	50	17	4.98	0.2	Adequate	
11	MI	3.8	11	Wet	Oral syrup	55	20	7.55	0.1	Adequate	
12	FI	2.4	12	Neurological	Injectable	84	15	19.67	0.2	Adequate	10 mg/kg x8 wks, then 15 mg/kg x4 wks
13	FI	2.5	6	Wet	Oral syrup	6	10	0.34	27.7	Adequate	Maropitant + flebocortid; diarrhoea on administration
14	FOHE	2.5	60	Dry + Neurological	Capsules	10	40	1.9	18.6	Unsure	
15	FOHE	2.9	11	Dry	Oral syrup	10	13.7	4.10	0.40	Unsure	
16	MC	3	8	Ocular + Neurological	Oral syrup	10	40	20.73	77.2	Unsure	Inflammation and fever; diarrhoea on administration
17	MC	3.6	108	Wet	Injectable	12	13.7	6.1	64.2	Unsure	

BW: body weight; ND: not determined; MC: male castrated; FI: female intact; FOHE: female ovariohysterectomized; MI: male intact.

 https://doi.org/10.3390/ani16121851

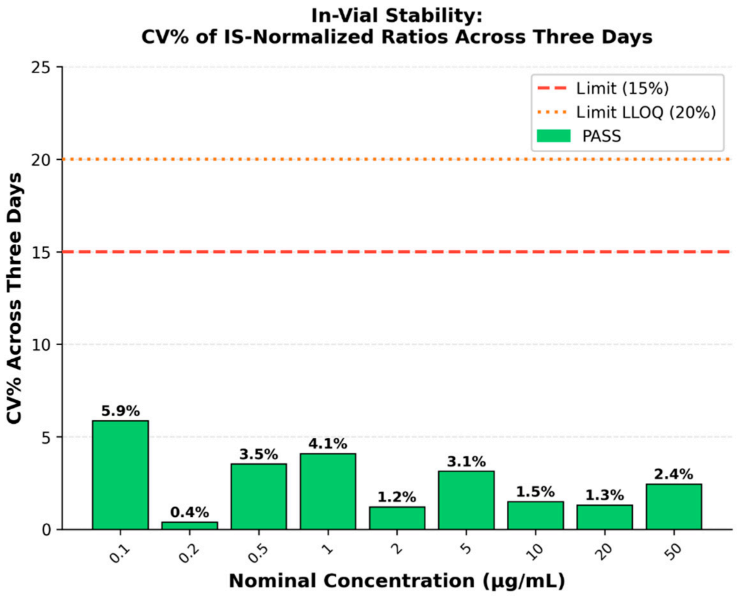

Figure S1. Processed sample stability assessed by CV% of IS-normalized response ratios across three independent analytical runs (days 1, 4, and 7) for extracted calibration standards maintained at 4°C in the autosampler. Dashed red line: acceptance limit (CV ≤15%); dotted orange line: acceptance limit at LLOQ (CV ≤20%). All concentration levels met acceptance criteria.

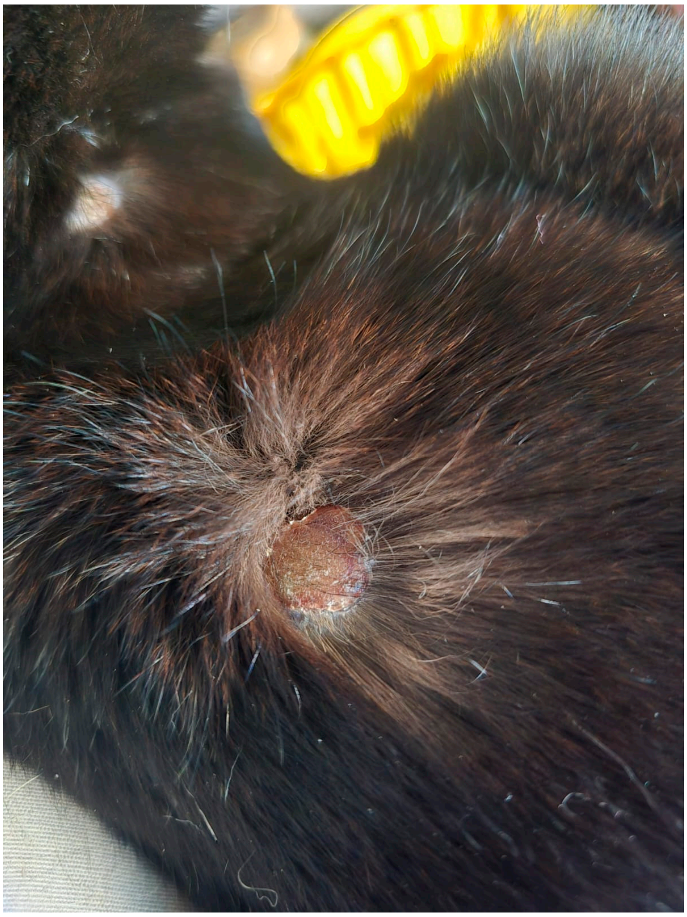

Figure S2. Ulcerated subcutaneous lesions at the injection site in cat 17. These lesions developed during the course of GS-441524 injectable treatment and may represent a source of secondary systemic inflammation, potentially confounding the interpretation of concurrent SAA concentrations.
